# Gene architecture is a determinant of the transcriptional response to bulky DNA damages

**DOI:** 10.26508/lsa.202302328

**Published:** 2024-01-02

**Authors:** May Merav, Elnatan M Bitensky, Elisheva E Heilbrun, Tamar Hacohen, Ayala Kirshenbaum, Hadar Golan-Berman, Yuval Cohen, Sheera Adar

**Affiliations:** Department of Microbiology and Molecular Genetics, Institute for Medical Research Israel Canada, Faculty of Medicine, Hebrew University of Jerusalem , Ein Kerem, Jerusalem, Israel

## Abstract

Bulky DNA damages block transcription and compromise genome integrity and function. The cellular response to these damages includes global transcription shutdown. Still, active transcription is necessary for transcription-coupled repair and for induction of damage-response genes. To uncover common features of a general bulky DNA damage response, and to identify response-related transcripts that are expressed despite damage, we performed a systematic RNA-seq study comparing the transcriptional response to three independent damage-inducing agents: UV, the chemotherapy cisplatin, and benzo[a]pyrene, a component of cigarette smoke. Reduction in gene expression after damage was associated with higher damage rates, longer gene length, and low GC content. We identified genes with relatively higher expression after all three damage treatments, including *NR4A2*, a potential novel damage-response transcription factor. Up-regulated genes exhibit higher exon content that is associated with preferential repair, which could enable rapid damage removal and transcription restoration. The attenuated response to BPDE highlights that not all bulky damages elicit the same response. These findings frame gene architecture as a major determinant of the transcriptional response that is hardwired into the human genome.

## Abstract (precis)

A comparative genomic study of the transcriptomic response to bulky DNA damages uncovers novel response genes and the features of their structure that allow them to be better repaired and transcribed.

## Introduction

Bulky, or helix distorting, DNA damages block transcription and replication, compromising cell function and survival. These include carcinogenic damages such as the ultraviolet (UV) radiation-induced cyclobutyl pyrimidine dimer (CPD) and (6-4) pyrimidine-pyrimidone photoproduct ([6-4]PP), damages induced by benzo[a]pyrene (BaP), a byproduct of smoking, and adducts induced by the chemotherapy drug Cis-diamminedichloroplatinum (cisplatin) (Friedberg et al, 2006). Beyond the different chemical structure of their damages, these agents target different nucleotide bases. UV damages occur in pyrimidine dimers, whereas cisplatin and BaP damages occur primarily in guanine (G) bases (Friedberg et al, 2006).

In human cells, bulky DNA damages are repaired by nucleotide excision repair (NER) (Spivak, 2015; Sancar, 2016; Zhang et al, 2022). The process of repair is divided into three major steps: (1) recognition of the damage, which can occur either directly (in general repair) or by a stalled RNA polymerase in a transcription-coupled manner (in transcription-coupled repair); (2) incision 3′ and 5′ of the damage, removing a nucleotide stretch of 24–32 nucleotides and leaving a single-stranded gap; (3) gap-filling DNA synthesis and ligation to restore intact double-stranded DNA.

There is a complex relationship between helix-distorting damages and transcription. These damages directly block RNA polymerases (Mayne & Lehmann, 1982; Nieto Moreno et al, 2023), but at the same time induce a transcriptional stress response (Herrlich et al, 1994; Blackford & Jackson, 2017). In parallel, active transcription enhances the ability to recognize and repair damages (Bohr et al, 1985; Geijer & Marteijn, 2018; Gaul & Svejstrup, 2021; van den Heuvel et al, 2021).

The transcriptional response to UV irradiation has been extensively studied (Geijer & Marteijn, 2018; Gaul & Svejstrup, 2021; van den Heuvel et al, 2021). After irradiation, transcription is shut down and recovers after ∼24 h, but only if the damages have been successfully removed (Mayne & Lehmann, 1982). Before shut-down and immediately after UV exposure, the bulk of promoter-proximal RNA polymerase II molecules are released into active elongation, possibly as a scanning mechanism for damages (Lavigne et al, 2017; Liakos et al, 2020; Bouvier et al, 2021). Recently, it was found that the catalytic Rpb1 subunit of RNA polymerase II stalled at damage sites is ubiquitinated at a single K1268 site (Nakazawa et al, 2020; Tufegdzic Vidakovic et al, 2020). This ubiquitination results in RNA Pol II degradation and is necessary for proper transcriptional shutdown.

At the same time, cells also trigger checkpoint responses through the ATR kinase, activating the p53 transcription factor and inducing the expression of early response genes, such as *ATF3*, *JUN*, and *FOS* (Herrlich et al, 1994; Shiloh, 2003; Blackford & Jackson, 2017). These early response genes are relatively short, possibly allowing them to evade damage. Indeed, longer genes that have higher propensities for damage are more strongly affected by UV (McKay et al, 2004; Andrade-Lima et al, 2015; Williamson et al, 2017).

To date, the study of transcriptional responses to bulky lesions focused almost exclusively on UV photoproducts and was conducted by multiple labs using different cell types and experimental methods (Andrade-Lima et al, 2015; Lavigne et al, 2017; Williamson et al, 2017; Tufegdzic Vidakovic et al, 2020; Bouvier et al, 2021). The global transcriptional shutdown induced directly by polymerase-blocking damages in cis, and by cellular factors in trans, has made the identification of a general set of up-regulated transcripts involved in an actively regulated transcriptional response more challenging. Therefore, our understanding of the transcriptional DNA damage response to bulky damages is still lacking.

Here, we hypothesize that a regulated transcriptional response to bulky DNA damages will be shared by additional RNA polymerase-blocking lesions beyond UV and by different cell types. Using a comparative RNA-seq approach and incorporating existing Damage-seq and XR-seq data, we characterize the shared features of genes with altered transcription after damage and identify novel candidate genes required for the bulky DNA damage response.

## Results

### Gene expression profiles cluster according to cell type and damage-inducing agents

**Figure 1.**
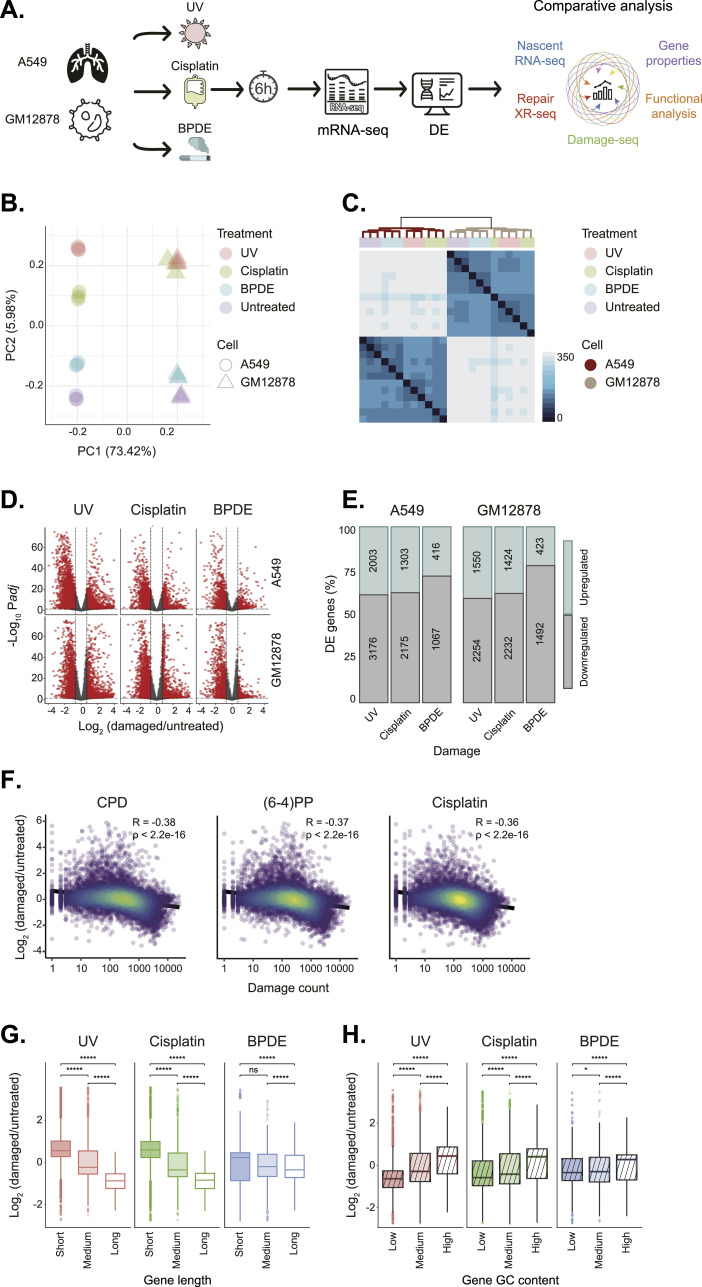
Determinants of expression after transcription-blocking damage. **(A)** Experimental outline. Human GM12878 or A549 cell lines were treated or untreated with transcription-blocking damage and incubated for 6 h before mRNA-seq. Comparative analysis was performed incorporating published Damage-seq and XR-seq data. DE, differential expression. **(B)** Principal component analysis of the gene expression data. **(C)** Heirarchial K-means clustering of the gene expression data. **(D)** Differentially expressed genes compared with control in A549 (top) or GM12878 cells (bottom) after UV, cisplatin, or BPDE treatment. In red: genes with *P*adj < 0.05 and log_2_ fold change > 0.7. **(E)** Percent of up-regulated (blue) or down-regulated (gray) genes under each condition compared with control. The precise number of genes in indicated within the bars. **(F)** Scatter plot and Spearman correlation between damage levels on the transcribed strand and the change in gene expression after treatment for UV and cisplatin damage in GM12878 cells. **(G)** Log_2_ fold change in gene expression after treatment in short (<14,590 bp), medium (14,590–51,570 bp), and long (>51,570) genes in GM12878 cells. **(H)** Log_2_ fold change in gene expression after treatment in genes with low (<41.6%), medium (41.6–49.5%), and high (>49.5%) GC levels in GM12878 cells. Boxes represent range between 25th and 75th percentile, and the line represents the median. ****P < 0.0001, *P < 0.05, ns: non-significant, based on Wilcoxon signed-rank test with Bonferroni correction.

**Figure S1.**
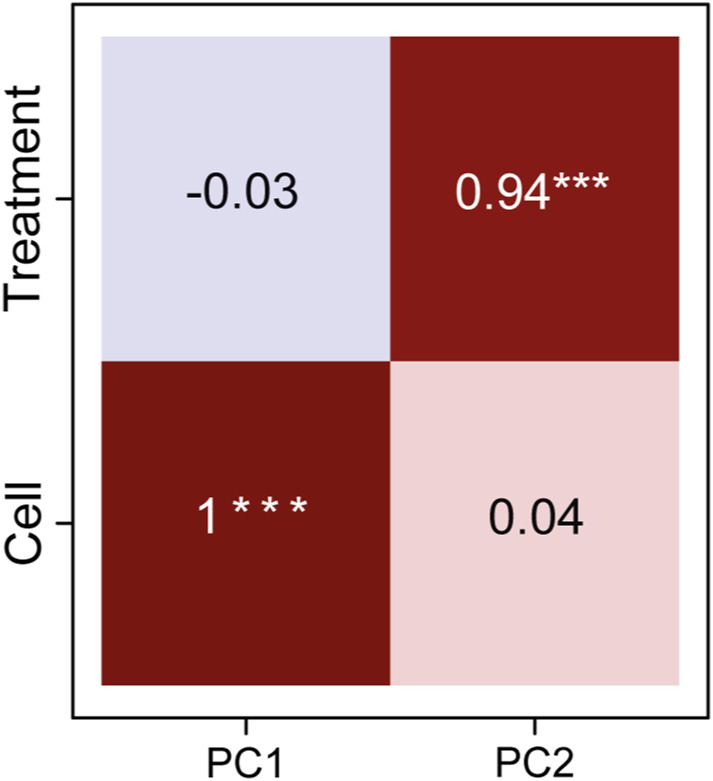
Statistically significant Pearson correlation coefficients between main principal components (PC1, PC2) and experiment metadata variables. Principal components (PC) PC1 and PC2 carry, respectively, 73.42% and 5.98% of the variance of the data. We observed a perfect positive correlation (+1) between PC1 and the cell line variable. Therefore, most of the variance in the data can be explained by the different cell lines. The second principal component, PC2, is mainly dependent on the treatment variable (0.94 Pearson correlation coefficient). Hence, it distinguishes between the three different bulky damages and the untreated samples.

For our comparative analysis of the transcriptional response to damage, we treated two starkly different cell lines, the GM12878 immortalized lymphoblast cell line and the A549 lung cancer cell line, with three different damage-inducing agents: ultraviolet radiation (UVC, 254 nm, 20 J/m^2^), benzo(a)pyrene diol epoxide (BPDE, 5 μM), and cisplatin (200 μM), and compared their transcriptional profile with an untreated control (Fig 1A). All experiments were performed with three biological replicates. We performed mRNA-seq 6 h after damage treatment to follow altered gene expression compared with the untreated control. Comparing both cell lines and treatments facilitates the exclusion of genes whose differential expression is dependent solely on the type of cell or damaging agent. Principal component analysis revealed that samples from the same cell line and treatment were clustered together, indicating both cell-related and treatment-related expression profiles (Figs 1B and S1).

Hierarchical clustering using the pairwise Euclidean distance between samples is consistent with the principal component analysis and shows that the two cell types form distinct clusters. Within each cell type, UV- and cisplatin-treated samples form a sub-cluster, whereas BPDE treatment appears closer to the untreated samples to form another sub-cluster (Fig 1C).

### Damage abundance, gene length, and GC content correlate to gene expression changes after genotoxic stress

Differential gene expression analysis indicated a general reduction in mRNA gene expression level as down-regulated genes were more prevalent compared with up-regulated genes for all damaging treatments (Fig 1D and E). This result is consistent with the previously reported global transcriptional shutdown in response to UV (Mayne & Lehmann, 1982; Geijer & Marteijn, 2018; Gaul & Svejstrup, 2021; van den Heuvel et al, 2021). In addition, we observed the scale of transcriptome changes differed between the bulky lesions. Whereas UV and cisplatin treatments led to strong transcriptional changes with >3,500 differentially expressed genes (DEGs) in each cell line, BPDE exposure only resulted in 1,483 and 1,915 DEGs in A549 and GM12878, respectively.

Because of the transcription-blocking nature of these lesions, we analyzed UV induced (6-4)PP and CPD and cisplatin damage abundances at genes based on previously published Damage-seq data from GM12878 cell line ([Hu et al, 2016; Hu et al, 2017], Fig 1F). In Damage-seq, the damaged base is identified at single nucleotide resolution based on its ability to block high-fidelity DNA polymerases in vitro. High-resolution initial BPDE-adduct mapping data are still not available. Increased lesion abundance is correlated with reduced mRNA levels after both UV and cisplatin treatment, indicating damage formation on the transcribed strand is a major determinant of differential expression.

**Figure S2.**
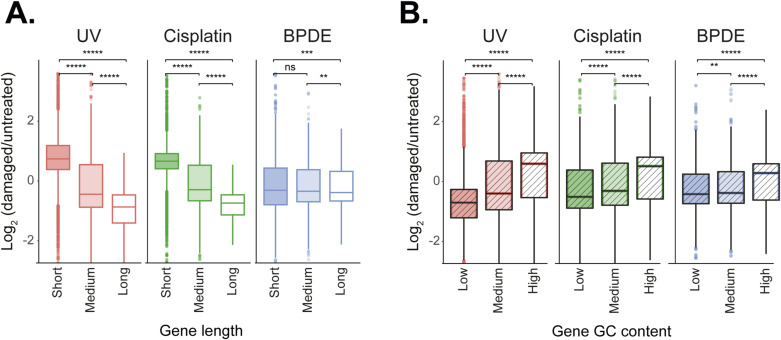
Determinants of expression after transcription-blocking damage in A549 cells. **(A)** Log_2_ fold change in gene expression after treatment in short (<14,590 bp), medium (14,590–51,570 bp), and long (>51,570 bp) genes in A549 cells. **(B)** Log_2_ fold change in gene expression after treatment in genes with low (<41.6%), medium (41.6%–49.5%), and high (>49.5%) GC levels in A549 cells. Boxes represent range between 75th and 25th percentile, and the line represents the median****P < 0.0001, *P < 0.05, ns, non-significant, based on Wilcoxon signed-rank test with Bonferroni correction.

Genome-wide mapping of UV and cisplatin damage formation indicates it is dictated primarily by the frequency of the targeted nucleotides (pyrimidine dimers for UV, G’s for cisplatin) and is distributed relatively homogeneously over the genome (Teng et al, 2011; Hu et al, 2016, 2017; Mao et al, 2018; Heilbrun et al, 2021). Thus, longer genes are statistically prone to contain more damages and their transcription likely to be more affected. This was previously shown for UV treatment (McKay et al, 2004; Andrade-Lima et al, 2015; Williamson et al, 2017). To evaluate the role of gene length in the transcriptional response to cisplatin and BPDE, we compared changes in gene expression after damage between short (up to 14,590 bp), medium-length (14,590–51,570 bp), and long genes (51,570 and over). For all three damage types, reduced expression was stronger in longer genes in both A549 and GM12878 cell lines (Figs 1G and S2A). As in the previous analyses of the transcriptional changes and the number of DEGs (Fig 1B–E), the response to BPDE was more attenuated compared with the response to UV and cisplatin.

UV damages occur primarily in pyrimidine dimers, whereas cisplatin and BPDE target primarily G nucleotides (Friedberg et al, 2006). We examined the relationship between the GC content of genes and their expression after damage induction. Genes were divided into those with low (up to 41.6%), medium (41.6%–49.5%), and high (49.5% and over) GC content. We found a similar significant correlation between GC content and expression after all three damage treatments. GC-poor genes had significantly reduced expression after damaging treatments in GM12878 and A549 cells (Figs 1H and S2B). Given that UV targets different nucleotides than cisplatin or BPDE, the shared correlation of expression to GC content for all treatments can not be explained by the frequency of target nucleotides but reflects an additional feature of the affected genes.

### Identification of novel damage-response factors

**Figure 2.**
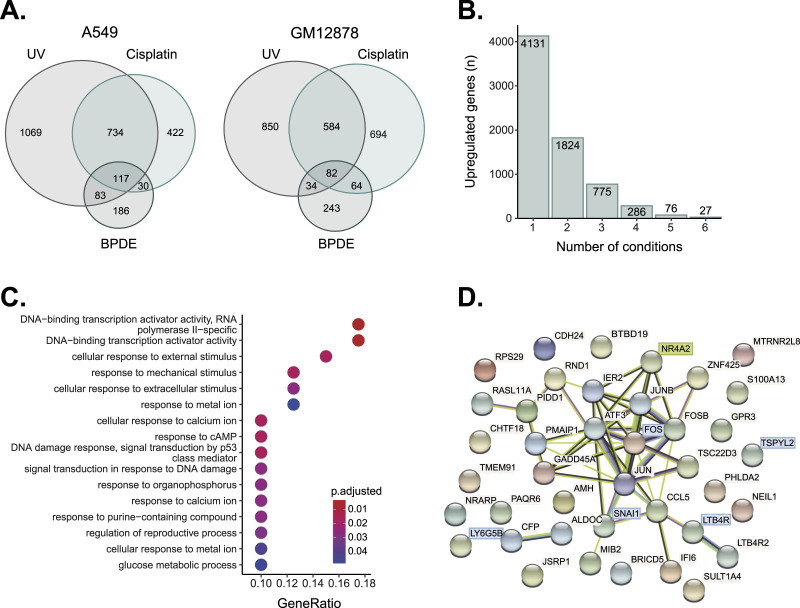
Defining genes consistently up-regulated after bulky DNA damage. **(A)** Venn diagram of the number of shared up-regulated genes after UV, cisplatin, and BPDE treatment in A549 (left) or GM12878 (right) cells. **(B)** The number of up-regulated genes shared in one to six of the conditions. The set of 76 genes enriched in five of the six conditions was taken for further analysis of consistently up-regulated genes. **(C)** Gene ontology terms associated with the consistently up-regulated genes. **(D)** Protein–protein interaction network analysis generated by STRING for the consistently up-regulated genes. Highlighted are genes we focus on in the following sections.

To identify genes that may serve a functional role in the response to damage, we focused on genes that had a relatively higher expression level after damage. We identified 117 and 82 genes in A549 and GM12878 cells, respectively, which exhibited higher relative expression across all (UV, cisplatin, and BPDE) treatments (Fig 2A). Under these cutoff conditions (|log2FC| ≥ 0.7 and *P*adj ≤ 0.05), 27 of these genes were shared in both cell lines (Fig 2B and Table S1). Of these, ten were protein-coding, including the well-known AP-1 damage response mediator components *FOS*, *FOSB*, and *JUN* and related inflammation mediators *CCL5*, *LTB4R*, *LTB4R2* (Herrlich et al, 1994; Appay & Rowland-Jones, 2001; Shiloh, 2003; Tager & Luster, 2003; Blackford & Jackson, 2017). Because this stringent requirement for up-regulation in six of the six conditions only contained ten mostly well-known damage-responsive protein-coding genes, we expanded our analysis of “consistently up-regulated genes” to include 49 additional protein-coding genes with higher expression in five of six combinations of three treatments and two cell types (a total of 76 genes, Fig 2B and Table S2).

Table S1 List of the consistently up-regulated genes shared in all six conditions. Genes up-regulated (log_2_FC > 0.7, *P*adj < 0.05) after UV, cisplatin, and BPDE in both A549 and GM12878 cells.

Table S2 List of the consistently up-regulated genes shared in at least five of the six conditions. Genes up-regulated (log_2_FC > 0.7, *P*adj < 0.05) after at least five of the six conditions: UV, cisplatin, or BPDE treatment in A549 or GM12878 cells.

To gain insight into the functions of the damage-responsive genes, we carried out gene ontology (GO) analysis (Fig 2C). Up-regulated genes were classified as involved in “DNA-binding transcription activator activity” and “DNA-binding transcription activator activity-RNA polymerase II-specific.” Fundamental cellular processes such as cellular response to external, mechanical, and extracellular stimuli and the response to DNA damage and ions were also inferred.

For 40 protein-coding, constitutively up-regulated genes, we performed STRING interaction network analysis (Fig 2D [Szklarczyk et al, 2015]). This analysis clearly identifies the key immediate early response genes *ATF3*, *FOS*, *FOSB*, *JUN*, and *JUNB* (*P*-value 7.12 × 10^−13^). It also identified *NR4A2* (*Nurr1*) which is defined as an immediate early response gene (Maxwell & Muscat, 2006; Herring et al, 2019; Safe & Karki, 2021), but has not been extensively implicated in DNA damage response.

**Figure 3.**
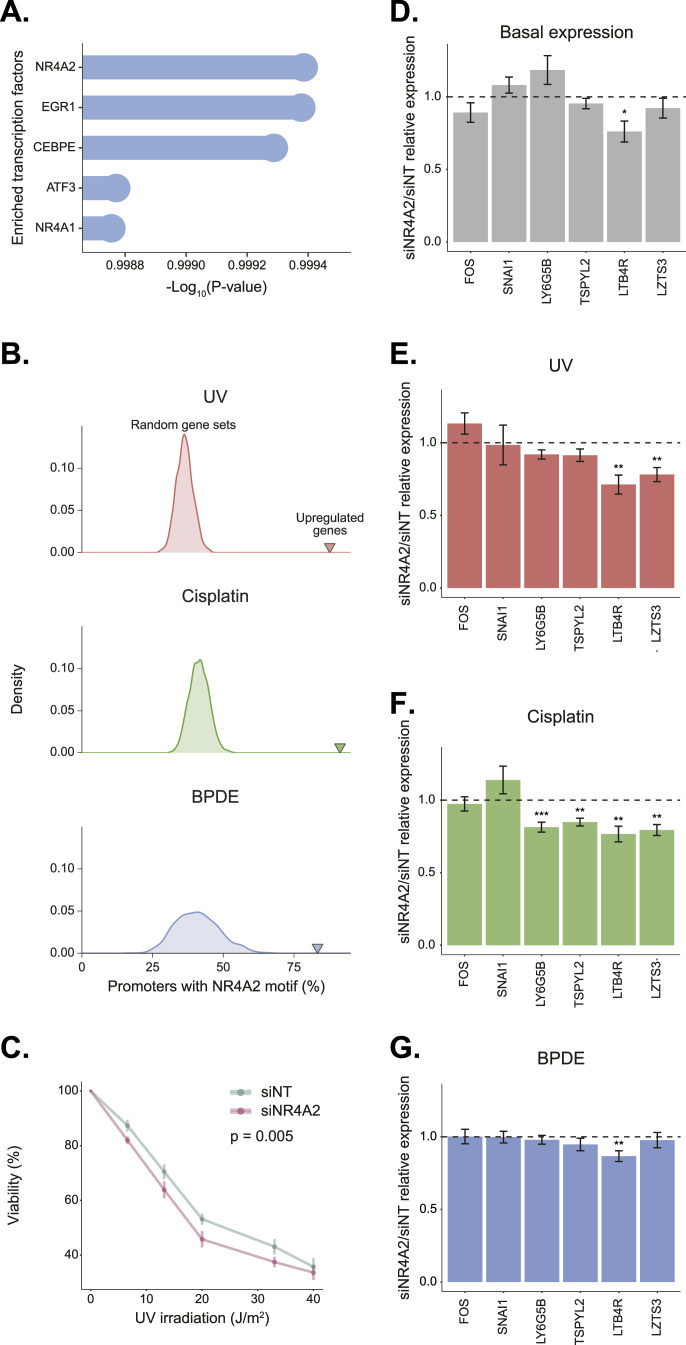
Identification of NR4A2 as a damage-responsive transcription factor. **(A)** Top scaled transcription factor ranks from ChEA3 for the genes up-regulated in all six conditions. **(B)** Analysis of the enrichment of NR4A2 motif in promoters of up-regulated genes. Histograms represent the distribution of the percent of random promoters with NR4A2 motif in 1000 iterations. Arrows indicate the percent of observed promoters with NR4A2 motif in the genes up-regulated in both A549 and GM12878 (For UV 612 genes, for cisplatin 519 genes, for BPDE 103 genes were analyzed). *P*-value < 0.001 in all three damage treatments. **(C)** Enhanced UV-sensitivity of NR4A2 knockdown cells compared with cells treated with non-targeting (GFP) siRNA control. Each data point represents three to six replicates. *P* = 0.0252 based on two-way ANOVA test. **(D)** Ratio of relative gene expression between siNR4A2 and siNT treated cells. Results are based on quantitative real-time PCR with n ≥ 4 replicates. Expression was normalized to the GAPDH housekeeping gene. **(E, F, G)** Same as in (D), but after UV (20 J/m^2^, (E)), cisplatin (200 μM, (F)) or BPDE (5 μM, (G)) treatments. *P < 0.05 based on unpaired one-tailed *t* test. NT, non-targeting.

**Figure S3.**
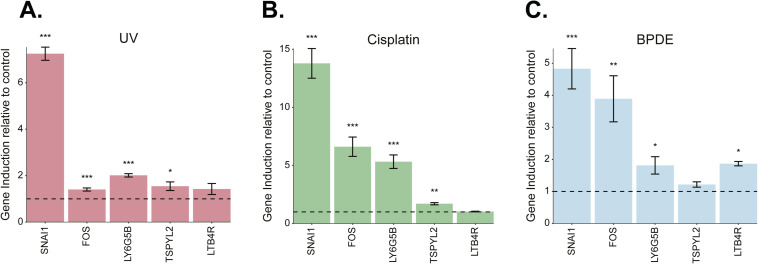
Consistently up-regulated genes are also up-regulated in 293T cells. Induction of a set of consistently up-regulated genes identified in this study in 293T cells using quantitative real-time PCR. **(A, B, C)** Cells were treated with UV (20 J/m^2^, (A)), cisplatin (200 μM, (B)), or BPDE (5 μM, (C)). Expression was normalized to GAPDH and compared with a non-treated control. six replicates. ***P < 0.001, **P < 0.01, *P < 0.05, unpaired one-tailed *t* test.

**Figure S4.**
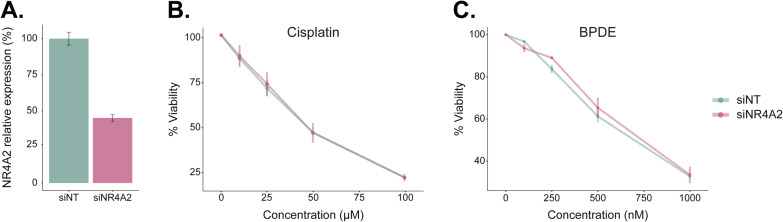
Knockdown of NR4A2 does not sensitize cells to cisplatin or BPDE. **(A)** NR4A2 expression measured in 293T cells treated with siRNA targeting NR4A2 or GFP as a non-targeting (NT) control. TBP was used as housekeeping gene for real-time PCR normalization. **(B)** Similar cisplatin-sensitivity of NR4A2 knockdown cells compared with non-targeting control. **(C)** Same as B except for BPDE treatment.

We used ChEA3, a tool for transcription factor enrichment analysis by orthogonal omics integration (Keenan et al, 2019), to identify candidate transcription factors involved in shared regulation of the up-regulated gene sets. For this, we used the most stringent cutoff, analyzing the 27 genes up-regulated in both cell lines and in all three damage treatments. The top predicted transcription factor involved with an integrated scaled rank of 6.143 × 10^−4^ was NR4A2 (Fig 3A). *NR4A2* is itself up-regulated after all treatments except BPDE in GM12878. The NR4A2 motif is indeed highly enriched in promoters of up-regulated genes after each one of the treatments in both cell lines (Fig 3B and Tables S3, S4, and S5). To test whether NR4A2 was involved in the response to bulky DNA damages, we knocked down the *NR4A2* gene. Knockdown was performed in 293T cells that are amenable to transfection. Before knockdown, we validated that five of our identified consistently up-regulated genes were mostly induced in the 293T cells in response to UV, cisplatin, and BPDE, highlighting the robustness of this response (Fig S3A–C). Cells with NR4A2 depletion were more sensitive to UV, suggesting this gene is required for efficient damage response (Figs 3C and S4A). NR4A2 knockdown, however, did not increase sensitivity to cisplatin or BPDE damage (Fig S4B and C). We tested the effect of NR4A2 on the expression of a set of candidate target genes after bulky DNA damage (Fig 3D–G). These included the early response gene *FOS* and four less characterized genes that we identified as consistently up-regulated: *SNAI1*, *LY6G5B*, *TSPYL2*, and *LTB4R*. We also tested the UV-induced *LZTS3* which is one of NR4A2’s top codependencies in the DepMap CRISPR screen data (Tsherniak et al, 2017). NR4A2 knockdown did not affect the basal expression of all tested genes except *LTB4R*, which was reduced. The expression of *LY6G5B*, *TSPYL2*, *LTB4R*, and *LZTS3* was reduced after UV or cisplatin treatments in NR4A2 knockdown cells compared with cells treated with non-targeting siRNA. We did not observe a significant effect of NR4A2 knockdown on the expression of any of the genes except *LTB4R* after BPDE treatment, which is in line with the comparatively attenuated response to this damage. Together, these results indicate the NR4A2 transcription factor plays a role in the transcriptional response to UV and cisplatin damage.

Table S3 List of the genes up-regulated after UV. Genes up-regulated (log_2_FC > 0.7, *P*adj < 0.05) after UV treatment in both A549 and GM12878 cells.

Table S4 List of the genes up-regulated after cisplatin. Genes up-regulated (log_2_FC > 0.7, *P*adj < 0.05) after cisplatin treatment in both A549 and GM12878 cells.

Table S5 List of the genes up-regulated after BPDE. Genes up-regulated (log_2_FC > 0.7, *P*adj < 0.05) after BPDE treatment in both A549 and GM12878 cells.

### Preferential repair of genes that are up-regulated after damage

**Figure 4.**
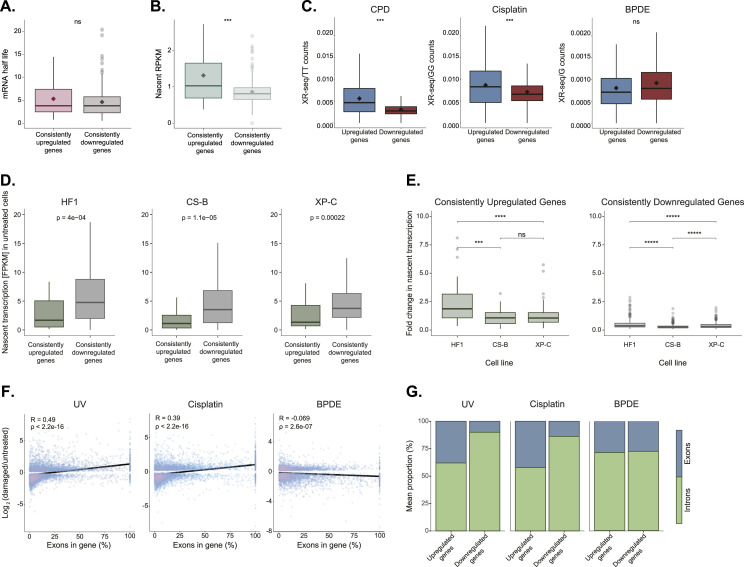
Higher exon content and enhanced repair of up-regulated genes. **(A)** The mRNA half-life based on Shi et al (2021) of the consistently up-regulated (n = 76) versus consistently down-regulated (n = 300) genes. **(B)** Nascent RNA transcription after UV treatment based on Bouvier et al (2021) of the consistently up-regulated (n = 76) versus consistently down-regulated (n = 300) genes. **(C)** Normalized XR-seq read count for UV (left), cisplatin (middle), and BPDE (right) in all the genes up-regulated (blue) or down-regulated (red) after each type of damage treatment. **(D)** Basal nascent RNA transcription (based on Andrade-Lima et al [2015]) of the consistently up-regulated (n = 76) versus consistently down-regulated (n = 300) genes in NER proficient (HF1, left), transcription-coupled NER deficient (CS-B, middle), and global genome NER deficient (XP-C, right) primary human fibroblasts. **(E)** Fold changes in nascent RNA transcription (based on Andrade-Lima et al [2015]) of consistently up-regulated (green) or consistently down-regulated (gray) genes obtained 6 h post-UV irradiation in NER proficient or deficient cell lines. **(F)** Correlation between exon content and changes in gene expression after damage. Spearman correlation coefficients are indicated for each damage type. **(G)** Average exon (blue) and intron (green) composition for up-regulated (left) and down-regulated (right) genes after UV, Cisplatin or BPDE treatments. ****P < 0.0001, *P < 0.05, ns, non-significant, based on Wilcoxon signed-rank test with Bonferroni correction.

**Figure S5.**
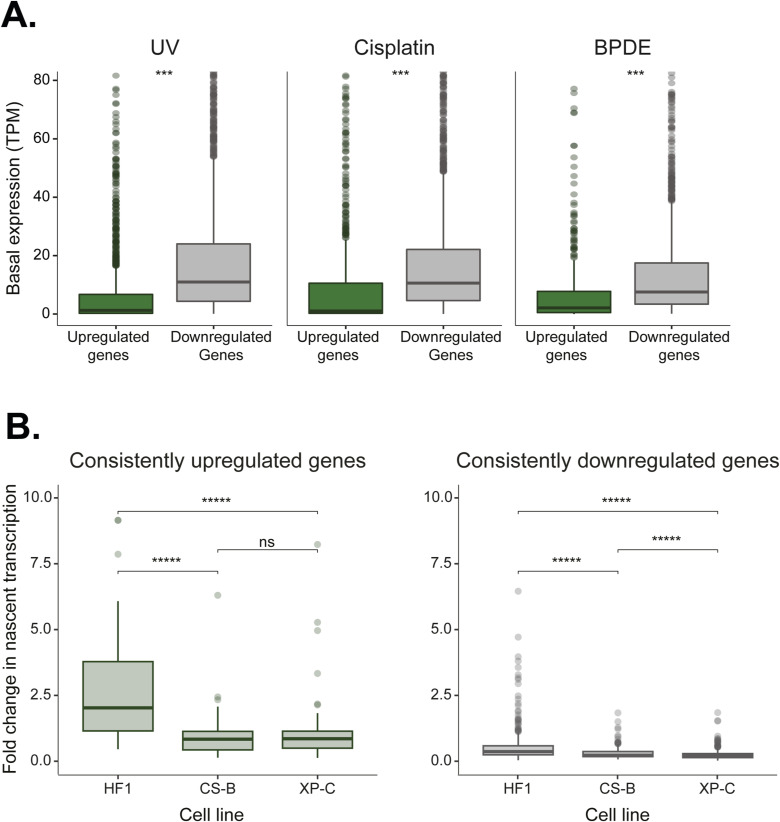
Lower basal gene expression in up-regulated genes. **(A)** Basal gene expression (TPM) in GM12878 for up-regulated (green) and down-regulated (gray) genes after UV (left), cisplatin (middle), and BPDE (right) after each damage treatment. **(B)** Fold changes in nascent RNA transcription (based on Andrade-Lima et al [2015]) of consistently up-regulated (green) or consistently down-regulated (gray) genes obtained 2 h post-UVC irradiation in NER proficient or deficient cell lines.

The level of a given mRNA transcript in cells is a function of both its nascent transcription rate and its stability. While most of the gene transcription is shut down, genes identified with higher relative expression may either be more highly transcribed or their transcripts may be more stable. We analyzed RNA stability data from A549 cells, which was measured by inhibiting transcription and performing RNA-seq at subsequent time points (Shi et al, 2021). Our set of 76 constitutively up-regulated genes did not display higher RNA half-lives (Fig 4A). However, based on nascent RNA transcription after UV in HeLa cells (Bouvier et al, 2021), the median nascent RNA levels of these genes were higher than the median transcription across a set of consistently down-regulated genes (shared in five of the six conditions, Fig 4B). This suggests their higher levels are the product of active or enhanced transcription, despite the damage. Transcription of these genes can only occur if they do not contain RNA-polymerase-blocking damages. This could be accomplished by lower damage susceptibility, but it could also be achieved by efficient NER. For each damage treatment, we identified the set of genes that are up-regulated in GM12878 cells, in which published XR-seq repair data are available (Hu et al, 2016; Li et al, 2017). In XR-seq, the excised oligos released during NER are captured and sequenced, providing high-resolution snapshots of repair. For UV and cisplatin damage, the up-regulated genes were associated with elevated repair efficiency compared with down-regulated genes. BPDE treatment did not yield significant differences in repair between the gene groups (Fig 4C and Table S6). This association could indicate that higher expression is driving higher transcription-coupled repair. Alternatively, higher repair efficiency could be enabling higher transcription. To discriminate between these options, we analyzed previously published basal and UV-induced nascent transcription in normal compared with repair-defective human skin fibroblast cell lines (Andrade-Lima et al, 2015). The repair-deficient cell lines were either defective in global genome repair (XP-C cells) or transcription-coupled repair (CS-B cells). The basal expression of our set of constitutively up-regulated genes before damage was actually lower than that of the down-regulated genes both in the nascent transcription data and in our RNA-seq data (Figs 4D and S5A). However, after UV, in the normal HF1 cells, the expression of these genes increased. This increase is not seen in the repair-deficient cell lines, indicating repair is necessary for the transcriptional response, and not vice versa (Figs 4E and S5B).

Table S6 Differential expression measurements for all analyzed genes in the study. The log_2_FC and *P*adj value for changes in expression of each gene under each of the conditions (UV, cisplatin, or BPDE treatments in A549 or GM12878 cells) compared with the untreated matching control cells.

We have previously shown that NER is more efficient in exons compared with introns (Heilbrun et al, 2021). When a higher proportion of the gene length is occupied by exons, we would expect the genes to have higher repair. Indeed, for UV and cisplatin treatment, we observed a positive correlation between exon composition and changes in expression after damage (Fig 4F). Exons comprised 37.97% and 42.16% of the damage-up-regulated genes, versus 9.99% and 13.71% in the down-regulated genes after UV and cisplatin, respectively (Fig 4G). In BPDE-treated cells, where we did not observe a statistically significant difference in repair between the up- and down-regulated genes, we also do not observe a difference in exon composition. Thus, the higher repair rates in the up-regulated genes may be attributed to the hardwired gene structure composed of higher exon and lower intron contributions.

## Discussion

Bulky DNA damages block RNA polymerases, eliciting a transcriptional shut-down that persists in human cells for ∼24 h. Most characterized in this respect is the response to UV radiation (Geijer & Marteijn, 2018; Gaul & Svejstrup, 2021; van den Heuvel et al, 2021). As opposed to chemical treatments, which require 1–2 h to elicit damages, UV damage is relatively instantaneous, facilitating accurate kinetic measurements. To define a general bulky DNA response, we performed a comparative RNA-seq study of the response to UV, cisplatin, and BPDE treatments and identified features that are common to all three damage types. Consistent with previous reports on UV damage (Mayne & Lehmann, 1982; Geijer & Marteijn, 2018; Gaul & Svejstrup, 2021; van den Heuvel et al, 2021), we found damage treatment resulted primarily in reduction in gene expression and that longer genes prone to higher damage levels were more strongly reduced. We also found a negative association between GC content and gene expression after damage. Specific damages form in specific nucleotide bases. UV damages occur primarily in pyrimidines (specifically TpTs) and would be higher in low-GC content genes, whereas cisplatin and BPDE damages occur primarily in G nucleotides and would be lower in low-GC content genes (Friedberg et al, 2006). However, the relationship to GC content is shared between all three damages, indicating it is not driven by the propensity to form damages directly but rather as an intrinsic characteristic of these genes. Low-GC content has been associated with higher transcription rate, which is reduced after damage (Veloso et al, 2014). Thus, one hypothesis would be that genes with higher initial transcription rates are more strongly affected by damage.

Though generally showing the same trends as UV and cisplatin, BPDE treatment consistently resulted in a weaker effect, and we did not find an association between expression after BPDE and repair efficiency. This could be attributed to lower damage frequencies obtained in the BPDE treatment we used, or additional effects of BPDE on cellular proteins and functions. These differences highlight that one cannot assume that the response to UV is shared by all bulky damages.

In parallel to the transcriptional shut-down, certain transcripts are necessary for the DNA damage response. Identification of these transcripts using differential expression analysis is complicated as the expression of most genes is reduced. Indeed, initial analysis of the data showed the RNA-seq samples clustered primarily by cell type and only then by damage treatment (Fig 1). We hypothesized that transcripts necessary for a general transcriptional response would be common to the three damaging treatments and to two very distinct cell types: GM12878 lymphoblasts and A549 lung adenocarcinoma cell lines. Our comparative RNA-seq approach identified sets of genes that were shared under all or most of the damage-induction conditions. These include the known early response genes previously reported to be induced after damage (Herrlich et al, 1994; Shiloh, 2003; Blackford & Jackson, 2017), and also novel candidate DNA response genes.

We identified less-characterized response genes associated with the early response network such as *NR4A2* (Maxwell & Muscat, 2006; Herring et al, 2019; Safe & Karki, 2021), but also genes that are not known interactors but that have been implicated previously in damage response. For example, the DNA glycosylase NEIL1, CHTF18 that is involved in loading DNA polymerase POLE at the sites of NER (Ogi et al, 2010), and TSPYL2 that regulates SIRT1 and p300 activity in response to DNA damage (Magni et al, 2019).

NR4A2 is an intriguing factor in the bulky DNA damage response that was not only consistently up-regulated but identified as a candidate transcription factor for regulating the damage response. NR4A2 is a member of the highly conserved nuclear receptor 4 (NR4) subgroup of nuclear receptors (Maxwell & Muscat, 2006; Herring et al, 2019; Safe & Karki, 2021) which are orphan receptors with currently no identified ligands. Though involved in diverse functions including proliferation and apoptosis, they are not significantly associated with the DNA damage response. We found knocking down NR4A2 resulted in enhanced UV sensitivity. This is consistent with a report by Jagirdar et al (2013) showing NR4A2 is recruited to repair foci. We also found that NR4A2 indeed appears to regulate the damage-induced expression of a subset of the genes tested, at least in response to UV and cisplatin. Together, these results point to a role of NR4A2 in the transcriptional response to bulky DNA damages, similar to ATF3, JUN, and FOS with which it interacts (Herrlich et al, 1994; Shiloh, 2003; Blackford & Jackson, 2017). Future work into this interesting new damage-response factor could elucidate precisely the gene set it regulates and whether it facilitates their transcription directly or indirectly.

There is an internal dissonance in the bulky damage response. On the one hand, transcription is shut down. On the other, transcription is necessary for induction of specific response genes. DNA response genes are generally shorter. In addition, UV triggers alternative splicing of gene transcripts and selection of earlier polyadenylation sites, creating primarily shorter isoforms (Munoz et al, 2009; Devany et al, 2016; Williamson et al, 2017; Murphy & Kleiman, 2020). Shorter isoforms would be less prone to damage, facilitating their expression.

Here, we uncover an additional structural determinant of the response to damage: the proportion of exons in the genes. Because exons exhibit better NER efficiency than introns, genes with inherently lower intron proportions would result in overall higher efficiency of repair. Thus, like gene length, the proportion of exons could be hardwired in the gene structure of DNA damage response genes to facilitate the recovery of their expression after damage despite the transcriptional shutdown.

## Materials and Methods

### Cell culture

GM12878 lymphoblast cell line (Coriell Institute) was grown in RPMI 1640 Medium without phenol red (01-103-1A; Biological Industries) supplemented with 15% FBS (10270106; Gibco, Rhenium Research Laboratory Equipment Ltd.), 4 mM glutamine (03-020-1B; Biological Industries), and 100 U/ml penicillin, 0.25 mg/ml streptomycin (03-031-1B; Biological Industries). A549 (ATCC CCL-185) and 293T cells were grown in DMEM medium (01-055-1A; Biological Industries) supplemented with 10% FBS, 2 mM glutamine, 1 mM sodium pyruvate (03-042-1B; Biological Industries), 100 U/ml penicillin, and 0.25 mg/ml streptomycin. Mycoplasma was monitored every 3–4 mo.

### Damage treatment and RNA-seq

For damage treatment, A549 cells were grown to ∼70% confluence in 100 mm dishes, culture medium was removed, cells were washed once in PBS and treated with either UV or media containing cisplatin or BPDE. For GM12878, cells were grown in 75 cm^2^ flasks to 700,000 cells/ml and were treated in 5 ml media without phenol red in 10 cm plates for each condition. For UV experiments, cells were irradiated with 20 J/m^2^ of 254 nm UVC (UVP XX15S, 95-0042-09). For cisplatin treatment, cells were incubated with media containing cisplatin at 200 μM (031 30 25429 05; Pharmachemie BV, Teva group) and for BPDE at 5 μΜ (#477; MRIGlobal). Cells were incubated for 6 h after damage treatment. Total RNA was extracted using GENEzol TriRNA Pure Kit with DNaseI (GZXD; Geneaid). RNA quality was assessed using RNA ScreenTape (5067–5576; Agilent) on 4200 TapeStation; Agilent, all samples having 10.0 RINe score. RNA-seq library preparation was performed using the KAPA Biosystems stranded mRNA-seq kit (KR0960; KAPA Biosystems) starting with 1 μg total RNA according to manufacturer’s protocol. Quality and concentration were assessed using High-Sensitivity RNA ScreenTape Analysis (5067–5579; Agilent). Experiments were performed in triplicates for each condition, and all 24 libraries were pooled and sequenced in a single NextSeq 500 lane, producing at least 15 million single-end reads per sample (75 nt long).

### siRNA transfection and knockdown

Cells were seeded at a density of 1.5 × 10^5^ in six-well plates. After 24 h, NR4A2 siRNA or non-targeting siRNA (EHU008731 and EHUEGFP MISSION esiRNA; Sigma-Aldrich) were transfected using TransIT-X2 Dynamic Delivery System (MIR-6000; Mirus Bio) at a final concentration of 24 nM. After another 24 h, incubation medium was replaced. After a final 24-h incubation, knockdown was confirmed by real-time PCR.

### Cell viability assay

Knockdown cells were seeded in 96-well plates at 4000 cells per well. Wells were treated with different doses of UVC, cisplatin, or BPDE 24 h after seeding. Viability was measured 48 h post-treatment using CellTiter-Glo Luminescent Cell Viability Assay Kit (#G7571; Promega, Biological Industries) according to manufacturer’s instructions. Viability luminescent was measured using Cytation 3 Imaging Reader (BioTek).

### Real-time PCR

Total RNA was extracted with GENEzol TriRNA Pure Kit (GZXD; Geneaid). RNA concentration and quality were measured by NanoDrop 2000c (Thermo Fisher Scientific). The cDNA was prepared using qScript cDNA Synthesis Kit (95047; QuantaBio). Expression level of genes was determined by real-time PCR conducted on a Bio-Rad CFX96 or CFX384 systems using iTaq Universal SYBR Green Supermix (1725121; Bio-Rad). Gene expression was normalized to the housekeeping genes TBP or GAPDH. Primer sequences are provided in Table S7.

Table S7 Primer sequences used for quantitative real-time PCR.

### Differential gene expression analysis

Quantification of transcript expression was performed using Salmon (Patro et al, 2017) and GENECODE v36 human reference transcriptome. Expression was estimated in a bias-aware quantification, correcting for GC content (--gcBias). Transcript-level expression was then mapped to GENECODE GRCH38 v36 genes and processed using the DESeq2 package (Love et al, 2014) for normalization and differential expression analysis (Table S6). Up- and down-regulated genes were defined by *P*adj ≥ 0.05 and log_2_(fold change) greater than 0.7 or less than −0.7, respectively.

The numbers of sequence reads mapped to each exon were counted using featureCount (Liao et al, 2014) with -O option (allowMultiOverlap) and analyzed at the gene level. After removing genes that have an average read of 0.5 per millions of reads in UV-nonirradiated samples, the data count was analyzed using DESeq2 (Love et al, 2014) to calculate the normalized fold change between the UV-irradiated and UV-nonirradiated samples. For the numbers of reads mapped to each transcript, we used Cuffdiff2 (Trapnell et al, 2013), and low expressers (RPKM < 0.1) were removed for subsequent correlation analyses. The annotations of genes and transcripts were obtained from the GENCODE homepage.

### Functional analysis of genes

Gene set enrichment analysis was performed using the R packages Enrichplot, DOSE and Clusterprofiler (Yu et al, 2015; Wu et al, 2021).

### Comparative data analysis

Damage-seq data (Hu et al, 2016, 2017) of UV photoproducts (CPD and 6-4PP) and cisplatin in GM12878 cell line were obtained from GEO (GSE98025, GSE82213). Raw data were further processed to detect damage position as in Hu et al (2016, 2017). Processed reads were aligned to the Hg38 reference genome and filtered by the damage target sequence (over 90% passed). Damage levels at genes were calculated using bedtools coverage (Quinlan & Hall, 2010) in a strand-specific manner.

XR-seq genome-wide maps of NER for CPD, cisplatin, and UV in the GM12878 cell line (Hu et al, 2016; Li et al, 2017) were obtained from GEO (GSE82213, GSE97675). Raw data were processed and aligned to the hg38 reference genome as previously reported (Hu et al, 2015; Adar et al, 2016; Li et al, 2017).

RNA half-life measurements in A549 cells were obtained from Shi et al (2021). For nascent transcription after UV, Bru-seq data were obtained from HeLa cells 6.5 h after irradiation (Bouvier et al, 2021). Normalized nascent transcription for each gene was calculated as the ratio between baseline transcription of a gene and transcription level at 6.5 h post-irradiation (RPKM). Comparative analysis was performed using bedtools (Quinlan & Hall, 2010).

Bru-seq nascent transcription data (Andrade-Lima et al, 2015) for normal (HF1), global genome repair deficient (XP67TMA XP-C cells), and transcription-coupled repair deficient (CS1AN CS-B cells) primary human skin fibroblasts before and after UV irradiation were obtained from GEO (GSE65985). Fold changes in nascent transcription were calculated for each gene as the ratio between baseline transcription of a gene to its transcription level 2 or 6 h post-irradiation.

To identify transcription factor motif occurrence in gene promoters, promoters were defined as 3 Kb upstream of the gene start and extracted using the bedtools slop (Quinlan & Hall, 2010). To identify the occurrence of the motif within these promoters, we used the meme fimo command (Bailey et al, 2009). For statistical significance, we conducted the same analysis on 1000 iterations of a matching set of randomly selected promoters. *P*-values were calculated as the number of iterations (of 1000) in which the ratio of motif occurrence in the control set was equal to or larger than the ratio of motif occurrence in the test set.

## Data Availability

All raw and processed sequencing data generated in this study have been submitted to the NCBI Gene Expression Omnibus (GEO; https://www.ncbi.nlm.nih.gov/geo/) under accession number GSE235681.

## Supplementary Material

Reviewer comments
